# The oracle of Delphi 2.0: considering artificial intelligence as a challenging tool for the treatment of eating disorders

**DOI:** 10.1007/s40519-023-01579-8

**Published:** 2023-06-19

**Authors:** Giovanni Abbate-Daga, Alberto Taverna, Matteo Martini

**Affiliations:** 1 Department of Neuroscience, University of Turin, Via Cherasco 15, Turin, Italy; 2 ASL City of Turin, Via San Secondo 29, Turin, Italy

**Keywords:** Artificial Intelligence, Eating disorders, Anorexia nervosa, Treatment, Psychotherapy, Ethics

## Abstract

In this editorial, we discuss how the diffusion of Artificial Intelligence (AI)-based tools—such as the recently available conversational AIs—could impact and transform eating disorders (EDs) care. We try to envision the possible use of AI by individuals affected by EDs and by clinicians, in terms of prevention, support to treatment, and development of new and actually personalized treatment strategies. We then focus on how the introduction of AI into psychotherapy could either represent an element of disruption for the therapeutical relationship or be positively and creatively integrated into session and inter-sessional dynamics. As technological advancements open scenarios where anyone could have access to a personal and all-knowing “oracle”, the ability to formulate questions, individuals’ experiences, and the scientific rigor with which clinicians study them must remain at the center of our work. Ethical and legal issues about the use of AI are also considered.

Feeding and Eating Disorders (EDs) are rapidly increasing and constantly evolving metabolic–psychiatric disorders, also connected to social changes. These disorders are difficult to treat, and there is currently no grade A evidence for their care [1]. Therefore, the planning of new forms of intervention is a priority. As is well-known, EDs predominantly affect adolescents and young people: these age groups are the most exposed to and open to the influence of technologies that are evolving in our world at a speed unimaginable until a few years ago. For these reasons, the study of the use of new technological tools is mandatory in our research field. We are only at the beginning of an era that can change ED care in unexpected and exciting ways, but we should be mindful of the risks and ethical issues ahead. Similar to what was suggested about the web in an old article by our group published by EAWD [2], new technological tools can be “a cure or a curse” in the treatment. In a broader view, the field of mental health has been searching for innovative digital solutions to support individuals with mental disorders, developing a set of knowledge that can be labeled as digital mental health or digital psychiatry [3]. Currently, the most studied application that makes use of digital technologies provides therapist-guided cognitive behavioral therapy via the Internet (ICBT). In this treatment format, the patient has access to a controlled digital platform where therapeutic materials—such as texts, video or audio clips, and structured goals to enhance behavior change—are available. ICBT requires little or no therapist intervention, and few or no face-to-face appointments. The therapist is a remote guide and a point of reference. In a just published meta-analysis that included also RCTs assessing body dissatisfaction and bulimic symptoms, ICBT was shown to be non-inferior when compared to traditional face-to-face CBT [4].

In recent months, we have witnessed a surge of ever more powerful and complex—yet easily accessible—technological tools build upon models powered by Artificial Intelligence (AI). Simply put and avoiding technical definitions, AI can be conceived as the application of computational systems to synthesize vast amounts of information and generate new ideas and solutions that normally require Human Intelligence. In particular, the development of the so-called Large Language Models (LLM) has made it possible to interact with AIs in a conversational manner, for instance, by posing questions and receiving original and articulated answers on most topics. The availability to the greater public of such tools (such as ChatGPT, launched in November 2022) has aroused extensive political, economic, and moral debates on their use and possible future scenarios. Certainly, the forthcoming integration of AI into mental care practices is opening new perspectives for patients seeking assistance and recovery.

In this editorial, we will try to outline possible applications for individuals with EDs.

The application of AI in the field of EDs can be divided into two areas: (1) the application of AI to a large amount of data gathered either from a single patient or from groups of patients without the direct involvement of the patient himself, allowing for an expansion in the possibilities of epidemiologic, biological and psychosocial studies, and (2) the active use of AI-based tools during treatment and in the daily living environment. Both areas can lead to eventual therapeutic advances but can also present obstacles and ethical problems. From this perspective, it is crucial to approach the use of AI with caution, ensuring that it serves as an empowering tool rather than a replacement for human support and care.

In our opinion, the most radical novelty of AI is related to the second point and lies in the fact that the accessibility to AI through browsers and apps bypasses the therapeutic management of more defined, pre-planned, and controlled formats (as in the ICBT). The individual is now free to use the AI in her/his own way, generating flexible and unforeseen scenarios. AI could be thus used both for and against therapy, as well as in completely new ways of treatment. It is clear that the question of the boundary of the therapeutic setting is under discussion: this boundary will potentially become more blurred or perhaps overall more articulated and multi-dimensional. However, when the use of AI by patients will spread, it will certainly be more difficult to define what is appropriate in the therapeutic context and what is not. Above all, the researcher and the clinician must be aware of the possibilities and limits of AI, in order to support the patient in a shared and critical use, regardless of whether a traditional therapy is used or not. We, therefore, believe that it is necessary to analyze in detail some crucial aspects of the use of AI in the EDs field.

As a possibility of prevention, the use of AI by individuals who are not yet receiving treatment should be considered. AI can facilitate faster awareness of having symptoms typical of EDs or of being an individual at ultra-risk for EDs. Early detection is crucial in managing eating disorders effectively and can make a difference in the possibility of greater efficacy of the treatment. AI algorithms can analyze vast amounts of data, including social media posts, online searches, and even smartphone usage patterns, to identify early warning signs. By recognizing behavioral patterns and language cues, AI could alert users and maybe even inform healthcare professionals, enabling them to intervene at the earliest stages of the disorder. Such early detection can significantly improve the chances of successful treatment and recovery. Furthermore, AI could provide more accurate and appropriate information on treatment and how effective interventions are for EDs in a much faster and more articulated way than the normal internet search. A recent study demonstrated that AI produced accurate and reproducible responses to common questions related to bariatric surgery: 86.7% of 151 responses obtained by AI was rated “comprehensive” by Board-certified bariatric surgeons [5].

On the other hand, a subject who has recently developed EDs symptoms, but is not motivated for treatment, could receive a booster of the disorder by obtaining information on purging behaviors, senseless training programs, unbalanced diets, and tips directly from the AI. In fact, even though safety measures are put in place by developers to avoid access to potentially harmful information, questions asked obliquely can currently easily get around the obstacle. Physicians and psychotherapists need to cooperate in the development of AI models to enhance the learning machine’s ability not to provide harmful information. It is also the duty of practitioners and researchers to check AI for the risk of mistakes making the new versions increasingly reliable. On this topic, an important caveat is that current conversational AIs are sometimes prone to generate so-called AI hallucinations or delusions [6], i.e., they can provide false information and present it as true (e.g., providing seemingly correct references to clinical studies that have never been conducted). Considering this eventuality is particularly crucial in the context of use made by vulnerable individuals: for EDs sufferers, false information about weight, food, and calorie consumption can be fatally detrimental. As is well-known, good treatment for EDs begins with discussing and re-establishing correct information on metabolism and body, thus counteracting fake news and pathological beliefs.

Using AI during treatment has at least three potential advantages. First, AI could be a valid help both as support between treatment sessions when active stimuli arise (e.g., urge to eat, weight gain greater than expected, anxiety before and after meals, etc.), and as an instrument for problem-solving suggestions in the living environment when the individual tries to achieve treatment goals (e.g., the agreed weekly weight gain goal). AI-powered devices could also monitor clinical parameters providing further information to be integrated with the evaluation of the progress of the overall treatment. Second, AI could help individuals confide their issues openly and freely. In fact, one of the primary challenges faced by individuals with eating disorders is the feeling of isolation and shame [7]. AI can step in as a non-judgmental companion, providing consistent and compassionate support. Through chatbots or virtual assistants, AI could engage patients in empathetic conversations, offering guidance, encouragement, and coping strategies. By simulating therapeutic interaction, AI could bridge the gap between therapy sessions, ensuring that individuals have access to support whenever they need it most, and could encourage the patient to confide their painful secrets to the therapist after having previously shared with the AI. Thirdly, AI could help in developing a very fine-grained treatment for any individual. AI has the potential to create care plans tailored to each individual’s unique needs. By analyzing vast datasets, including medical records, genetic information, and treatment outcomes, AI algorithms can generate insights and predictions (so called “digital phenotyping”). This data-driven approach could assist clinicians in developing more accurate and effective treatment strategies, leading to improved outcomes for patients with EDs. This exciting horizon is related to the wider use of AI in medicine: in fact, the analysis of large quantities of data in omics sciences through intelligent artificial systems is starting to give preliminary outstanding results [8]. Conversely, in the future, the use of AI by single individuals could make their personal and private tool co-evolve with their unmet needs, preferences, and difficulties, which AI could learn to know in an increasingly refined way over time. Lastly, AI could early detect irregular meal patterns and increased physical activity, reminding the patient of the risk of relapse.

Concerning psychotherapy research, some considerations are crucial. In addition to the possibilities of using an enormous amount of data to help the individual as described above, it is necessary to question and study with rigorous research protocols how AI modifies the psychiatrist/psychotherapist–patient relationship and how the new unpredictable AI–patient relationship can be. AI introduces a third (non)person in the treatment with consequent pros and cons. While AI may better support the patient, the patient may idealize AI and devalue the therapist. The essential point will remain the ability of the two human beings to keep the various technological and relational aspects interconnected and integrated with each other. Could we imagine the AI recording the sessions and detecting some relevant information lost during the previous sessions, so that the therapeutic couple can use them? Could we imagine that part of the sessions with the therapist will be focused on the projection on the AI of powerful, either positive or disgusting, hating feelings, like on a parent? In this sense, the well-known mechanisms of transference and empathetic mirroring could be modified in an unknown way both in relation to the therapist and to the AI itself. Psychotherapy is basically a journey together that goes through many ruptures and repairs of the therapeutic alliance. AI could fit into this dynamic like glue or scissors. In this area of research, two interesting studies about EDs from the ANTOP group—one of the best protocols on psychotherapy for Anorexia Nervosa—have highlighted how much the intersession process (i.e., the patients’ processing of psychotherapy between sessions) can be significant for the outcome [9, 10]. More specifically, studies show that higher levels of patients’ experiencing negative emotions when recalling therapeutic dialogue or thinking about therapy during dreaming/drowsy states can be a negative prognostic index. The research question of whether AI can mitigate or amplify this phenomenon is indeed intriguing. As partially outlined in a previous paper [11], several types of use of AI during psychotherapy can be foreseen: (a) human psychotherapy delivered, AI informed, (b) AI psychotherapy delivered, human supervised, (c) psychotherapy delivered only by AI, (d) AI used without prior agreement with the therapist, but discussed in session, and (e) AI used without the therapist knowing. Naturally, in the not-too-distant future, psychotherapists will need to be informed and specifically trained on the difficult task of the integration of AI into psychotherapy.

Last, but not least there are ethical considerations. Eating disorders also affect individuals who live in areas far from specialized centers and access to quality care can be a challenge due to geographical or financial barriers. As a positive effect, AI can provide accessible and affordable support. Mobile applications and online platforms powered by AI algorithms can deliver evidence-based alert signals, interventions, psychoeducation, and self-help resources to individuals, regardless of their location or financial means. This democratization of care can increase the reach of treatment options. However, many problems that are difficult to solve are also on the table. Privacy and data security can be serious problems that damage the rights of the person. This issue must be prioritized, ensuring that personal information remains confidential and protected. Transparency in AI algorithms is equally important, as patients should understand how their data is used. Specific laws and norms on data sharing in the use of AI are needed and urgent.

Finally, in case AI did not provide the solutions expected by a seriously suffering individual, it could be dangerous. In fact, the individual could drop out from treatment without the possibility for a clinician to be able to console and re-engage her/him or—in extreme cases—perceive the risk and actively intervene to protect the patient. Careful consideration of the avoidable drop-out risks (an occurrence unfortunately common in the field of EDs), and ultimately the suicidal risk, is necessary. However, given that AI is now available to everyone anyway, our mission is to study the phenomenon in the most honest, friendliest, and most in-depth way possible, involving the same people who suffer from EDs on the front line to minimize the risks of unknown use. Our duty is to help our patients understand how responsibly to use opportunities opened by AI, learning from them at the same time. It is better to ride the wave than to end up beneath it.

Obviously, AI should complement, not replace, human involvement, as the empathetic understanding and expertise of healthcare professionals are essential in establishing confidence and building therapeutic relationships. In fact, unfulfilled or unfounded emotional expectations towards AI could frustrate the patient and undermine trust in all forms of psychotherapy and in the therapist himself. Furthermore, non-verbal communication and embodied cognition are known to play pivotal roles in the therapeutic relationship, and we have the mandate to preserve the relationship and the person in contemporary psychiatry [12]. Although the application of AI tools can also be developed in the field of emotion recognition through detecting expressions of face and body [13], the interactivity and authentic exchange of a real relationship is far from reproducible. Currently, it remains unlikely that AI could have that implicit/explicit nuanced emotional capacity and could fine-tune that empathic response that a human can provide. In this sense, in coming months and years, clinicians and researchers operating in the field of mental health should indeed help in understanding and defining what is essential that remains “made by a human for a human”.

Everything discussed draws huge perspectives and raises huge doubts about the future of the treatment of EDs. New technological tools are making it possible to interact with and inquire into collective human knowledge in fascinating and thought-provoking ways. One of the challenges that lie ahead will be to help people suffering from EDs to formulate questions in an articulated and effective way thus developing the ability to gather useful information and insights while being always aware of the intrinsic limits to any answer. Already in ancient Greece, when consulting the oracle of Delphi, it was crucial to know how to read between the lines of the prophecy, as the answers could be ambiguous and point the wrong way. In ancient times like now, the role of human beings and their ability to “read” together the information remains at the basis of the possibility of having good solutions and of using our extraordinary self-correction skills. It will be a new bet poised between the Delphic sense of the limit and the Odyssean aspiration to overcome it.

In conclusion, the use of AI for eating disorder treatment represents an important and stimulating challenge for ED care. By providing new ways to facilitate the analysis of extraordinary data networks, facilitate early detection of symptoms, provide compassionate companionship, build personalized treatment plans, and make care accessible, AI has the potential to empower individuals with EDs in their recovery journey. However, a delicate balance must be maintained, ensuring that AI is used as a supportive tool within an ethical framework that upholds patient privacy and maintains the central role of human clinicians. Therefore, there is an urgent need to investigate the efficacy of AI through randomized controlled trials, to establish sound research methodology and guidelines for appropriate use, and to address the multiple ethical and legal issues associated with the growing diffusion of AI in therapies and in our lives. All stakeholders should be involved in this impressive social change: scientifically evaluating the impact of new AI practices on the relationship between clinician and patient is the decisive challenge for the coming years.

## Data Availability

The original answer by ChatGPT is available upon request.

## References

[1] Crone C, Fochtmann LJ, Attia E (2023). The american psychiatric association practice guideline for the treatment of patients with eating disorders. Am J Psychiatry.

[2] Abbate-Daga G, Gramaglia C, Pierò A, Fassino S (2006). Eating disorders and the internet: Cure and curse. Eat Weight Disord.

[3] Torous J, Bucci S, Bell IH (2021). The growing field of digital psychiatry: Current evidence and the future of apps, social media, chatbots, and virtual reality. World Psychiatry.

[4] Hedman-Lagerlöf E, Carlbring P, Svärdman F (2023). Therapist-supported internet-based cognitive behaviour therapy yields similar effects as face-to-face therapy for psychiatric and somatic disorders: An updated systematic review and meta-analysis. World Psychiatry.

[5] Samaan JS, Yeo YH, Rajeev N (2023). Assessing the accuracy of responses by the language model chatGPT to questions regarding bariatric surgery. Obes Surg.

[6] Deng J, Lin Y (2023). The benefits and challenges of ChatGPT: an overview. Front Comput Intell Syst.

[7] Panero M, Longo P, De Bacco C (2022). Shame, guilt, and self-consciousness in anorexia nervosa. J Clin Med.

[8] Liu G, Catacutan DB, Rathod K (2023). Deep learning-guided discovery of an antibiotic targeting *Acinetobacter baumannii*. Nat Chem Biol.

[9] Hartmann A, Zeeck A, Herzog W (2016). The intersession process in psychotherapy for anorexia nervosa: characteristics and relation to outcome. J Clin Psychol.

[10] Zeeck A, Hartmann A, Wild B (2018). How do patients with anorexia nervosa "process" psychotherapy between sessions? A comparison of cognitive-behavioral and psychodynamic interventions. Psychother Res.

[11] Miner AS, Shah N, Bullock KD, Arnow BA, Bailenson J, Hancock J (2019). Key considerations for incorporating conversational AI in psychotherapy. Front Psychiatry.

[12] Gabbard GO (2018). Preserving the person in contemporary psychiatry. Psychiatric Clinics.

[13] Lott LL, Spengler FB, Stächele T (2022). EmBody/EmFace as a new open tool to assess emotion recognition from body and face expressions. Sci Rep.

